# Effects of Dietary Supplementation with Carrot-Derived Rhamnogalacturonan-I (cRG-I) on Accelerated Protective Immune Responses and Quality of Life in Healthy Volunteers Challenged with Rhinovirus in a Randomized Trial

**DOI:** 10.3390/nu14204258

**Published:** 2022-10-12

**Authors:** Sue McKay, Annemarie Teitsma-Jansen, Esther Floris, Tamara Dekker, Barbara Smids, Ridha Khurshid, Wim Calame, Alwine Kardinaal, René Lutter, Ruud Albers

**Affiliations:** 1 NutriLeads B.V., Bronland 12-N, 6708 WH Wageningen, The Netherlands; 2 Amsterdam UMC, Department of Experimental Immunology, University of Amsterdam and Amsterdam Infection & Immunity Institute, Meibergdreef 9, 1105 AZ Amsterdam, The Netherlands; 3 NIZO, Kernhemseweg 2, 6718 ZB Ede, The Netherlands; 4 StatistiCal B.V., Strandwal 148, 2241 MN Wassenaar, The Netherlands

**Keywords:** quality of life, rhinovirus-16, common cold, healthy adults, innate immune, RG-I, WURSS-21, Jackson score

## Abstract

An adequate and balanced supply of nutrients is essential for maintaining health, and an optimal immune response is fast, contained and properly controlled, curbing infections quickly while minimizing damage. Several micronutrients contribute to normal immune function and certain dietary fibers, for example pectic polysaccharides, can play an important role in educating and regulating immune cell responses. The aim of this paper is to elaborate on our initial findings that dietary supplementation with carrot-derived rhamnogalacturonan-I (cRG-I) accelerates and augments local innate immune and anti-viral interferon response to a rhinovirus-16 (RV16) infection and reduces the severity and duration of symptoms in humans. Dietary intake of cRG-I also enhanced immune responses to this respiratory viral infection as measured by ex vivo stimulation of whole blood with the Toll-like receptor 3 (TLR3) ligand polyinosinic:polycytidylic acid and NK cell function. Consumption of cRG-I also reduced the negative effects of this common cold infection on quality of life as assessed by individual symptom scores. RG-I from carrot is a safe, sustainable, and economically viable solution that could easily be integrated into food products and dietary supplements aiming to support immune fitness and wellbeing.

## 1. Introduction

Good nutrition that provides an adequate and balanced supply of nutrients is essential for maintaining health. While certain vitamins (A, B6, B12, folate, C, D and E) and minerals (zinc, copper, selenium, and iron) contribute to the normal function of the immune system [1,2], other food components and especially certain fibers can play an important role in educating and regulating immune cell responsiveness. Everything we ingest is sampled by immune cells in the Waldeyer’s ring in the nasopharynx, in Peyer’s patches in the small intestine and by dendritic cells protruding through the intestinal epithelial layer [3]. The responsiveness of macrophages and dendritic cells (DC) that sense molecular patterns on microorganisms, but also on food components, via pattern recognition receptors (PRR) can be modulated and is known as immune training or priming. Moreover, changes in composition of the gut microbiota and production of bioactive metabolites such as short chain fatty acids (SCFA) can also modify immune responsiveness [4]. Given that macrophages and DCs from the intestine can recirculate via lymph and blood to other mucosal areas such as the respiratory tract, alterations in immune responsiveness due to effects in the gut can increase responsiveness to, e.g., airway infections [3].

Pectic polysaccharides have been reported to modulate immune responsiveness in the intestine via direct recognition by PRR as well as via stimulation of short chain fatty acid (SCFA) production by the microbiota [5,6]. Pectic rhamnogalacturonan I (RG-I) domains have been shown to modulate innate immune function as shown by the secretion of cytokines, chemokines, and reactive oxygen species, and by enhancing innate immune cell responses such as phagocytosis and natural killer cell activity, and by partially restoring the response to influenza vaccination in immunosuppressed mice [7,8,9,10,11]. They have also been shown to modulate the gene expression of intercellular adhesion molecule-1 (ICAM-1) which is a key docking molecule used by rhinoviruses to infect airway epithelial cells [12].

Recently we demonstrated that dietary intake of carrot-derived RG-I accelerates and augments the innate immune and anti-viral interferon responses to an infection with rhinovirus strain 16 (RV16) and reduces the duration and severity of symptoms in healthy volunteers [13].

Challenge studies with RV16 have been used extensively to better understand immune responses to rhinovirus and other respiratory viruses. Local immune responses to RV16 infection include the release of interferons and other pro-inflammatory mediators such as CXCL-10 and CXCL-8 leading to infiltration of inflammatory cells. Besides local responses, there are systemic responses including increased levels of T-helper and T-regulatory cell type cytokines [14] as well as an increase in plasma levels of CXCL-10 [15,16] and production of antibodies. RV16 challenge studies have also been employed to test the efficacy of various therapeutics [17]. However, they have rarely been used for dietary intervention studies even though infections challenges are uniquely suited to demonstrate clinically relevant effects as well as underlying changes in immune responsiveness due to food ingredients [18,19]. Rhinovirus infections account for most (50–70%) common colds with children becoming infected 8–12 times per year and adults 2–3 times per year [20,21,22,23]. Rhinoviruses, similar to coronaviruses, are group IV non-enveloped positive-sense single-stranded RNA (+ssRNA) viruses that generally infect and replicate in nasal and bronchial epithelial cells as well as macrophages causing respiratory infections in the upper airways. Moreover, they can impact the lower airways in susceptible individuals, e.g., newborns, elderly, and people with airway disorders [24,25]. RV16 infections usually resolve within 1–2 weeks and result in characteristic common cold symptoms including runny nose, sore and scratchy throat, cough, and tiredness. These symptoms can be reliably assessed using validated questionnaires and because the precise moment of the standardized infection is known when using a challenge model, the time course and magnitude of the defensive anti-viral immune responses can be analyzed in detail. 

Upon infection, RV16 attaches to ICAM-1, enters the host cells and hijacks the cellular machinery to replicate. During viral replication double-stranded (ds) RNA is formed and the anti-viral response is initiated by the recognition of RV16 and dsRNA by PRR such as Toll-like receptor (TLR) 2 on the cell surface, and TLR3, TLR7, TLR8, and retinoic acid-inducible gene I like (RIG-I) receptors in the infected cells. This response initiates the synthesis of interferons (IFN) [26,27,28,29]. Signaling of secreted IFNs via cell surface IFN receptors starts a cascade of intracellular pathways to promote the expression of hundreds of interferon-stimulated genes (ISGs) in infected and neighboring cells [30]. These ISGs encode proteins that inhibit virus replication and virus entry and stimulate early innate immune responses including the release of pro-inflammatory mediators to attract and activate circulating immune cells from the blood [25,28,31]. The infiltrating cells (neutrophils, dendritic cells (DC), macrophages, lymphocytes, eosinophils, etc.) also release pro- and anti-inflammatory mediators and play a role in killing infected cells, scavenging, antigen presentation to the adaptive immune system and ultimately resolution of the inflammatory response. The central IFN signaling pathways are strictly regulated to contain the anti-viral response while minimizing collateral damage from the destructive mechanisms that are deployed [27,32]. Importantly, the symptoms experienced during a rhinovirus infection, and most other viral infections, are not caused by the presence of the virus, but by the hosts immune response to the virus [33]. An optimal immune response is therefore fast, contained and properly controlled to curb the virus infection while minimizing damage, symptoms, and the impact on quality of life (QoL). 

There are a number of tools for assessing the common cold and the Jackson scale is often used to define and evaluate cold and flu symptoms, but it does not address QoL measures. The Wisconsin Upper Respiratory Symptom Survey (WURSS) was developed using subjects with Jackson-defined colds and it includes questions related to symptomatic as well as functional impairment. WURSS was specifically designed to be an evaluative outcomes instrument, and its use has been validated making it an ideal tool for investigating the impact of a dietary intervention on individual common cold symptoms as well as QoL attributes [34,35,36].

In this paper we elaborate on the initial findings that dietary intake of cRG-I accelerates innate immune responsiveness and reduces severity and duration of symptoms during a RV16 infection in humans [13]. We also zoom in on the individual symptoms and quality of life attributes affected and show that supplementation with cRG-I not only accelerates local immune responsiveness to the respiratory infection but can also modulate immune responsiveness of cells from the systemic compartment, measured following an ex vivo challenge. 

## 2. Materials and Methods

### 2.1. Study Design

**Figure 1:**
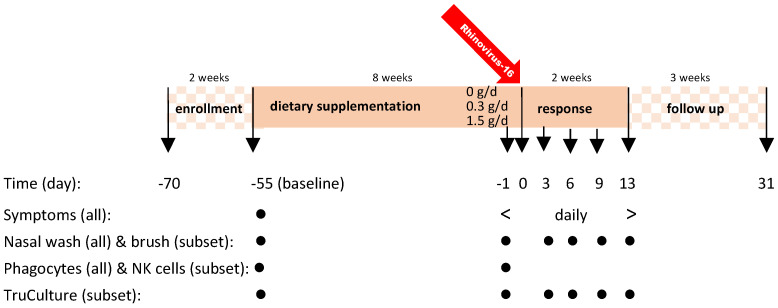
Study design and sampling moments. No dietary supplement taken during enrollment phase d-70 to d-55. Supplementation started on d-55 and continued through to d13. In the follow-up phase, without supplementation, safety parameters and seroconversion were assessed [13].

This single center, randomized, double-blind, placebo-controlled dose response study had three arms with 0, 0.3 g/day (d) and 1.5 g/d cRG-I in a parallel design. Details on design, adverse events, safety monitoring, disposition of subjects and primary outcome have been described earlier [13]. The study period comprised four periods: enrolment (screening, eligibility), dietary supplementation for 8 weeks, response to RV16 infection (exposure on day 0, d0) with continued supplementation for 2 weeks, and 3-week follow-up without supplementation (Figure 1).

The presence of neutralising RV16 antibodies excluded subject participation in this study. A throat swab on d-1 to detect natural respiratory viral infections by polymerase chain reaction (PCR) and an online Jackson questionnaire from d-7 till d-1 were used to determine whether subjects should be infected with RV16 [13]. Only subjects not suffering from a common cold (assessed by the Jackson questionnaire) or fever within 7 days prior to the viral challenge were eligible for RV16 inoculation. The presence of a cold was defined as two of the following three criteria being present (i) a cumulative Jackson symptom score of at least 14 over a 6-day period, (ii) the subjective impression of a cold by the volunteer and (iii) rhinorrhea on at least 3 days. Eligible subjects, who were symptom-free and PCR-negative, completed the Wisconsin Upper Respiratory Symptom Score-21 (WURSS-21) questionnaire, online, the day before infection (d-1) and then every day until d13, in the morning, and the Jackson questionnaire in the evening.

The WURSS-21 questionnaire provides a comprehensive set of validated questions that and is divided into 3 sections: (A) the total score, and “how do you feel today?” (item 1), (B) upper respiratory tract infection (URTI) symptoms (items 2–11) to rate the severity of cold symptoms over the last 24 h, and (C) quality of life (items 12–20) to assess the impact of a common cold on daily activities. In addition, item 21 “compared to yesterday, I feel that my cold is:” provides a relative indication of symptom severity. Sampling was performed as depicted in Figure 1. In a nested subset (16 participants selected randomly per group), nasal brushes were performed to collect nasal epithelial samples for transcriptome analyses at the same time intervals as for nasal lavage [37]. On d-55 and d-1 blood samples were collected to assess phagocytic activity, from the nested subset for natural killer cell activity, and from the same subset on d-55, d-1, d3, d6, d9 and d13 for analysis of immune responses in whole blood cultures stimulated with polyinosinic:polycytidylic acid (poly-IC: TruCulture^®^).

### 2.2. Healthy Participants

The study physician assessed participants’ health based on medical history and use of medication, and participants were between 18 and 65 years of age with a BMI between 18.5 and 30.0 kg/m^2^. Volunteers with a RV16 antibody titer > 1:6 at screening [37], with a medical history of hay fever, rhinosinusitis, asthma, COPD, other underlying pulmonary, cardiovascular, or auto-immune disease, or food allergy were excluded. A complete overview of inclusion and exclusion criteria, the minimal dietary restrictions and other relevant criteria were reported earlier [13].

### 2.3. Dietary cRG-I Supplementation

cRG-I is a natural extract from carrot (*Daucus carota* subsp. sativus) and was supplied by NutriLeads (Wageningen, The Netherlands). It is a water soluble non-digestible fermentable fiber enriched (80%) in the RG-I domain of pectin. The extraction method and extract characteristics (composition and structure) were described earlier [7]. The monosaccharide composition of cRG-I was (% mol/mol): 14.3 rhamnose; 34.8 arabinose; 19.6 galactose; 0.8 fucose; 4.3 glucose; 0.9 mannose; 0.7 xylose; 25.0 galacturonic acid. Participants were instructed to take the dietary supplement, supplied as a powder in a sachet, once a day with food and drink items of choice during the first meal (preferably breakfast).

### 2.4. Procedures

#### 2.4.1. Rhinovirus 16 Infection

Participants were challenged by instillation of 100 tissue-culture infectious doses (TCID50) RV16 (GMP-prepared RV16: UBiopred EU/IMI) in the nasal cavity, as described earlier [37].

#### 2.4.2. Nasal Brush and Nasal Lavage

The detailed nasal brush and nasal lavage procedures have been described in earlier publications [7,37]. Cells were separated from the nasal lavage fluid by centrifugation (10 min at 465× *g* at room temperature (RT)) then processed for cell differentiation using a cytospin. The supernatant was used to determine RV16 viral load by PCR [37] and soluble mediators using multiplex by Luminex. Sequential samples were analyzed batch-wise to limit variability and internal controls were used to verify consistency.

#### 2.4.3. Systemic Immune Responsiveness Assessed Using Blood Samples

##### Phagocytic Activity

The effect of dietary supplementation with cRG-I on phagocytic activity (d-55 and d-1) in whole blood was determined by measuring the percentage of phagocytes (granulocytes and monocytes) that phagocytosed fluorescently labelled *Ecoli*. FITC-labelled *Ecoli* was either (i) non-opsonized, (ii) opsonized with complement + IgG, or (iii) opsonized with IgG by incubating with serum or inactivated serum for 30 min, before mixing with blood cells. Mean fluorescence intensity (MFI) was measured after either 5, 15 or 30 min incubation, using a flow cytometer (detailed protocol in Supplementary Material).

##### Natural Killer (NK) Cell Activity 

The quantitative determination of NK cell function and activity (d-55 and d-1) was assessed in batches for logistic reasons using frozen PBMC, by measuring cytotoxicity and degranulation with a flow cytometer. Briefly, NK cells (effector cells, E) were incubated overnight with medium in the presence of IL-2 (0, 25, 100 and 400 U/mL). They were subsequently incubated with two ratios of K562 target (T) cells (2.5:1 and 5.0:1 E:T) for the cytotoxicity assay and at 0.1:1 E:T for the degranulation assay, for 3 h. The percentage of apoptotic target cells (K562) and CD107a (Biolegend, The Netherlands) positive cells was determined for cytotoxicity and degranulation, respectively. CD107a (also known as LAMP1) is a marker expressed on the NK cell surface after granule membranes have fused with the NK cell membrane [38].

##### Immune Profile in Whole Blood

Blood was drawn directly into pretreated poly-IC TruCulture^®^ tubes (Rules-Based Medicine, Austin, TX, USA) and processed according to manufacturer’s instructions [39]. The blood culture supernatants were then used to determine levels of CXCL-10, CXCL-8, CXCL-5, GM-CSF, IFNγ, IL-1β, IL-2, IL-6, IL-10, IL12p70, IL-13, IL-17, IL-23, and TNF-α using human cytokine OptiMAP (Rules-Based Medicine, Austin, TX, USA) and IFN-alpha was determined by ELISA (Mabtech, Stockholm, Sweden).

### 2.5. Statistical Analysis

The statistical approach has been detailed in the previous paper. Briefly, generalized estimating equations (GEE) analysis was used to evaluate potential dose-dependent effects. Confounding parameters were included, and the analysis was performed in a stepwise design with dose and the interaction between dose and time as independent parameters [13]. Comparison of baseline values between the various doses was performed via the parametric *t*-test.

Statistical analyses were conducted for the intention-to-treat (ITT) population and for some specifically defined subsets, the outcome for the per-protocol (PP) population was in agreement with that of the ITT population [13]. Presence of outliers was tested applying the Grubbs’ test. In some cases, a post hoc analysis was performed to quantify a difference in outcome. This was also performed via GEE modelling with confounding factors but then comparing two separate doses.

Throughout the study a *p*-value of 0.05 was considered to identify significance applying one-sided evaluation based on the expected outcome of the parameters analyzed. Statistical analysis was performed via Stata, version 12 (Statacorp, College Station, TX, USA) and GraphPad, version 6 (GraphPad Prism, San Diego, CA, USA).

## 3. Results

### 3.1. Study Subject Characteristics

One hundred and seventy-seven healthy adults (18–65 years) met the inclusion and exclusion criteria (representative of the general population) and were randomly assigned to receive 0, 0.3 or 1.5 g/d cRG-I as powdered dietary supplement. Subjects consumed one sachet of the dietary supplement daily with their breakfast for 8 weeks prior to infection and for a further 2 weeks during the response phase of the trial. A total of 146 subjects (ITT population) were intranasally exposed to an experimental infection with 100 tissue-culture infectious doses RV16. Of the infected participants, 48 were randomly selected to participate in the nested sub-study which involved additional measurements (subset). Disposition of subjects, demographic characteristics of the different (sub)populations, compliance, adverse events, and safety as well as initial outcomes are detailed in [13].

### 3.2. Detailed Analysis of the Effect of cRG-I on Perceivable Symptoms of RV16 Infection and Its Impact on Quality of Life

**Figure 2:**
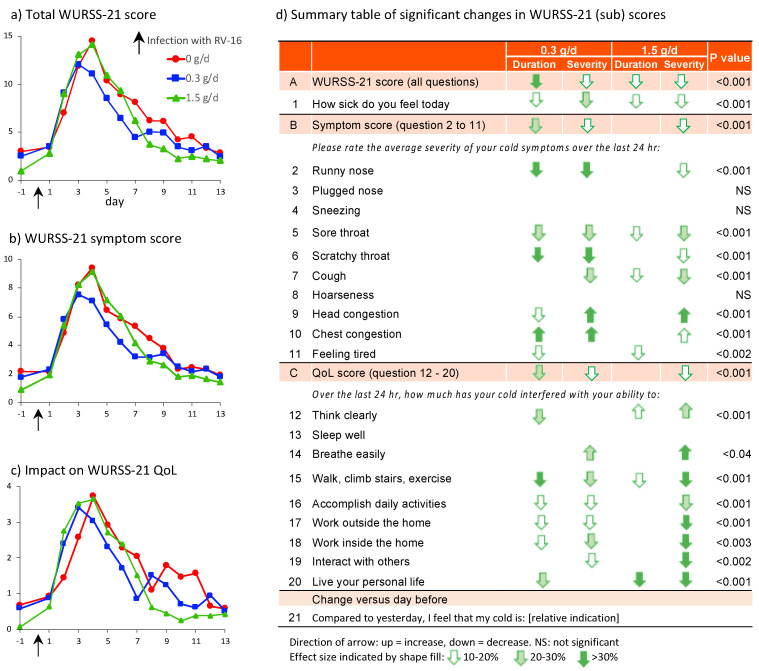
(**a**) Time course of WURSS-21 total score (*p* < 0.001), (**b**) symptom score (sum question 2–11) (*p* < 0.001) and (**c**) impact on QoL (sum question 12–20) (*p* < 0.01), (**d**) Summary table of individual items in validated WURSS-21 questionnaire indicating significance in GEE analyses per item across all doses over the full time course. The stronger beneficial impact on reducing the severity of QoL attributes in the 1.5 g/d group is mainly attributed to the response observed after d8 in a limited number of subjects. For significant items with changes > 10%, the direction of change is indicated for the 0.3 and 1.5 g/d group relative to the 0 g/d group.

The intention to treat (ITT) analysis of the total WURSS-21 score revealed a statistically significant time-dependent parabolic response for all groups (Figure 2a, *p* < 0.01) with a less severe response and shorter duration in the cRG-I groups. This resembles the effect of cRG-I on the first question “how sick do you feel today?” (Figure S1 in Supplementary Material) and the WURSS-21 symptom scores (Figure 2b), described earlier [13], which showed a peak response on d4 in the 0 g/d group. GEE analyses showed a statistically significant reduction in severity (lower peak value) and duration (shift of the curves to the left) in the cRG-I groups over the 13 days post infection. In the 0.3 g/d group the symptom score severity was reduced by 20% and duration by 25%.

The last column in Figure 2d shows that dietary supplementation with cRG-I, in all but a few cases, significantly reduced severity and/or duration of individual symptoms underlying the composite symptom score. In the 0.3 g/d group subjects reported more than 30% reduction in severity and duration of a runny nose and scratchy throat, 20–30% reduction in severity and duration of a sore throat, and a 20–30% reduction in cough severity. Interestingly, a 10–20% reduction in duration of head congestion but with an increase in severity as well as an increase in duration and severity of chest congestion was reported. A slightly lower impact on individual symptom scores was generally seen in the 1.5 g/d group, and a 10–20% reduction in duration of feeling tired was reported in both groups. RV16 used in the infection induces mild symptoms, with total scores averaging 10 (range 0 to 54) on a 70-point scale, but reflective of a natural common cold infection. In 10 volunteers the sum of symptom severity scores was above 40 (data not shown).

Although overall scores for the quality of life attributes were low and varied between subjects, they revealed a significant beneficial effect of cRG-I (Figure 2c). Dietary supplementation with cRG-I resulted in a significant reduction in severity and/or duration of most of the individual QoL scores (Figure 2d) with a total reduction in severity between 10% and 20% compared to the 0 g/d group.

**Figure 3:**
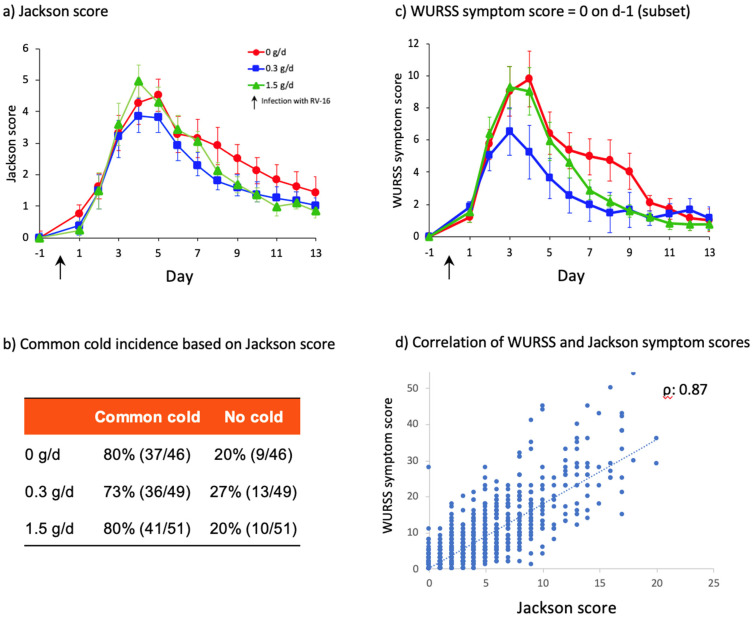
(**a**) Perceived symptoms following infection with RV16. Jackson score in full ITT population (*n* = 146), the GEE model showed a parabolic association in time as well as dose of cRG-I (*p* < 0.0001), (**b**) Common cold incidence based on established criteria for Jackson score (score at least 2 on at least two consecutive days, ITT population *n* = 146) no significant differences, (**c**) Subset with WURSS symptom score = 0 on day (d-1) prior to infection (*n* = 87) the GEE model showed a parabolic association in time as well as dose of cRG-I (*p* < 0.0001), (**d**) Correlation of WURSS-21 and Jackson symptom scores, analyzed using Spearman’s rank-correlation, coefficient *ρ*: 0.87, *p* < 0.0001, linear regression analysis indicates a positive association between the data of both scores.

To minimize the interference of concurrent natural infections, only study participants without ongoing respiratory viral infections were infected with RV16. Study participants completed a short Jackson questionnaire every day from d-6 until d-1 (before infection) and this was continued after infection (d0–13). The Jackson score is based on the sum of severity points comprising 8 questions with a 4-point scale (0–3) so maximum score amounts to 24 [40]. Figure 3a shows that after infection, the time course and effect of cRG-I reflected by the simpler Jackson score (reported in the evening) resemble the WURSS-21 symptom scores (Spearman’s rank-correlation coefficient *ρ*: 0.87, *p* < 0.0001, Figure 3d) (reported in the morning). The incidence of common cold, following RV16 exposure, based on the established criteria for a Jackson score of at least 2 on at least two consecutive days is shown for the 3 groups in Figure 3b. Interestingly, Figure 2a shows that some subjects had a WURSS-21 symptom score greater than 0 on the day prior to infection, even though only symptom-free (Jackson score), PCR-negative subjects were exposed to RV16. To investigate this further, a post hoc analysis of subjects with a WURSS-21 symptom score of 0 on the day before infection was performed, and a pharmacokinetic model was used to determine the symptom elimination half-life, as described earlier (Figure 3c) [13]. In this subgroup of 87 subjects the beneficial effect of cRG-I is even more pronounced than in the full ITT population, as is the finding that the effect is strongest in the 0.3 g/d group. Symptom severity (peak symptom score) was reduced by 33% and symptom duration was reduced by 43% in the 0.3 g/d group compared to the 0 g/d group. Of the 59 subjects with a WURSS symptom score above 0 on d 1, 18 had a score above 3 (range 4–18, average 7.7) and 7 included a positive score for question 1 (feeling sick). The other 41 subjects had low scores (typically 1) across the various questions with question 11 ‘feeling tired’ being scored most frequently (range 1–3, average 1.6). The Jackson questionnaire does not include a question related to tiredness.

### 3.3. Correlations between Perceived Symptoms and Biological Parameters

**Figure 4:**
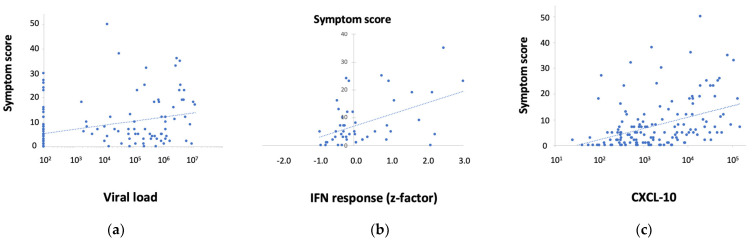
(**a**) Weak association between viral load and WURSS symptom scores on d3 (*p* < 0.003), (**b**) Association between IFN-response genes and WURSS symptom scores on d3 (*p* < 0.04), (**c**) Association between CXCL-10 in nasal lavage and WURSS symptom scores on d3 (*p* < 0.0001). Statistical analyses were performed using linear regression analysis, showing positive associations.

No strong correlations were found between any of the biological parameters and perceived symptoms. Figure 4a shows that viral load has a weak, but significant, association with WURSS symptom scores on day 3 (slope 1.54, *p* < 0.0003), no significant association was found on d6, 9 and 13 (not shown). Viral load did not correlate with any of the markers in nasal lavage. The expression of critical IFN response genes in nasal epithelium was measured in the nested subset (*n* = 16/group), the z-scores were significantly associated with symptoms on d3 only (slope 4.2, *p* < 0.04) (Figure 4b). Of the markers in nasal lavage, CXCL-10 showed the best association with symptoms on d3 (slope 4.26, *p* < 0.0001) and on d6 a weak association (slope 1.0, *p* < 0.006, Figure 4c). No associations were observed when assessing data across multiple days.

### 3.4. Effect of cRG-I on Systemic Immune Responses Prior to Infection

#### 3.4.1. Phagocytic Activity

The percentage of monocytes phagocytosing *Ecoli* and the MFI for granulocytes and especially monocytes was slightly higher on d-55 than d-1 in the cRG-I groups (Table S1 in Supplementary Material).

#### 3.4.2. Poly-IC Stimulated Whole Blood Assays

**Figure 5:**
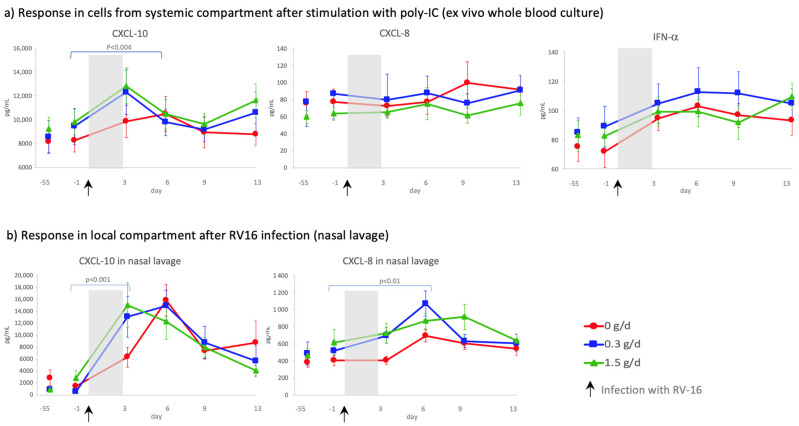
(**a**) Concentration of CXCL-10, CXCL-8 and IFN-α in the supernatant of whole blood cultures stimulated with the viral antigen poly-IC (nested subset). Each symbol represents mean ± SEM of minimal 12 subjects per group. (**b**) Concentration of CXCL-10 and CXCL-8 in nasal lavage at baseline (d-55), just before (d-1) and during rhinovirus infection (d3–13). Symbols represent means ± SEM of ITT population (*n* = 146). *p* values indicate the time interval after infection during which the groups are significantly different. Shaded area indicates time between infection and first biological marker sampling point.

Poly-IC (TLR3 agonist; dsRNA) stimulation of whole blood using the TruCulture^®^ system showed that dietary intake of cRG-I did not affect ex vivo production of inflammatory markers prior to an immune challenge with RV16, however a slight, but not significant, increase was observed in CXCL-10 in the cRG-I groups (Figure 5a, d-55 compared to d-1). In blood samples collected after the RV infection, PBMCs were more responsive to poly-IC and production of CXCL-10 (*p* < 0.002) and IFN-α (*p* < 0.001), but not CXCL-8, showed a significant time-dependent response to the RV16 infection. Figure 5a also shows that dietary supplementation with cRG-I significantly increased the CXCL 10 response to poly-IC stimulation across the time interval d0 to d3 (*p* < 0.004) after RV16 infection. A significant increase in CXCL-10 levels was also observed locally in the nasal compartment following exposure to RV16 (Figure 5b). Even though CXCL-5 (*p* < 0.004) and CXCL-13 (*p* < 0.04) levels were increased in a time-dependent manner in whole blood cultures after the RV infection, concentrations of TNF-α, IFNγ, IL-6 and IL12 p70 were not significantly affected (data not shown), and GM-CSF, IL-2, IL-10, IL-17 and IL-23 were (mostly) below the limit of detection in this culture system, none of these responses differed between cRG-I groups.

#### 3.4.3. Natural Killer Cell Activity

**Figure 6:**
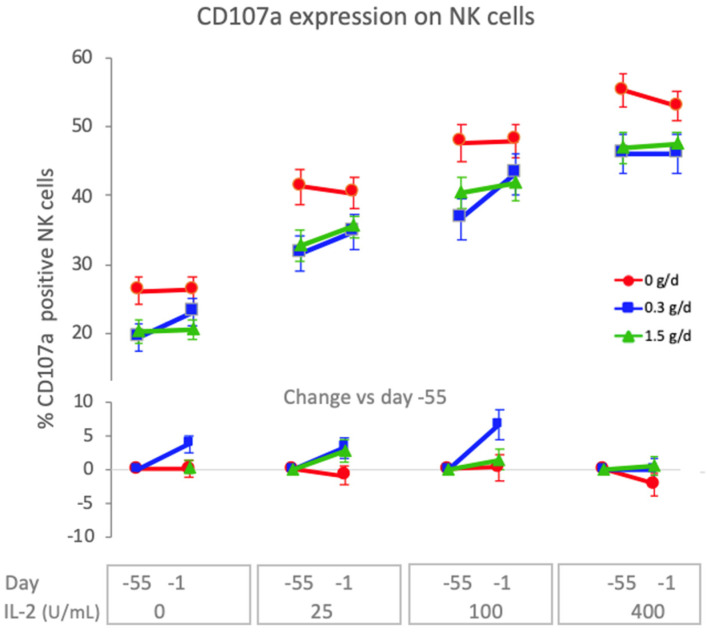
Expression of the activation marker CD107a on NK cells, after ex vivo incubation with K562 target cells, was significantly increased (*p* < 0.001) in the cRG-I groups. Increasing concentrations of exogenously added IL-2 reduced this effect (*p* < 0.03). Top: % of CD107a positive NK cells, and bottom: % change d-1 vs. d-55 (baseline), optimal for IL-2 concentrations 25–100 U/mL).

Natural killer cells are a subset of lymphocytes that play an important role in the early innate immune response to virus infections. They are activated in response to type I IFN and cytokines (IL-2, IL-12, IL-15 and others) and can kill virus-infected epithelial cells and secrete mediators (IFN-γ, GM-CSF and others) to regulate innate and adaptive immune responses. To assess the effect of 8 weeks dietary supplementation with cRG-I on natural killer cell activity before the RV16 infection, expression of CD107a and cytotoxic activity were measured on d-55 and d-1 following ex vivo incubation of PBMC as a function of exogenous IL-2 in the presence of K562 target cells. Figure 6 shows that CD107a (also known as LAMP-1), which is an intracellular membrane marker detected on the surface of cytotoxic lymphocytes following degranulation, was significantly increased in both cRG-I groups compared to the placebo, with a more pronounced effect in the 0.3 g/d group (*p* < 0.001). This effect was significantly reduced (*p* < 0.03) when the amount of exogenously added IL-2 increased indicating that the cRG-I effect remained within the biologically regulated range (Figure 6). The cytotoxic capacity of the NK cells to kill K562 target cells was not significantly affected.

### 3.5. Effect of cRG-I on the Kinetics of Local and Systemic Innate Anti-Viral Immune Responses 

**Figure 7:**
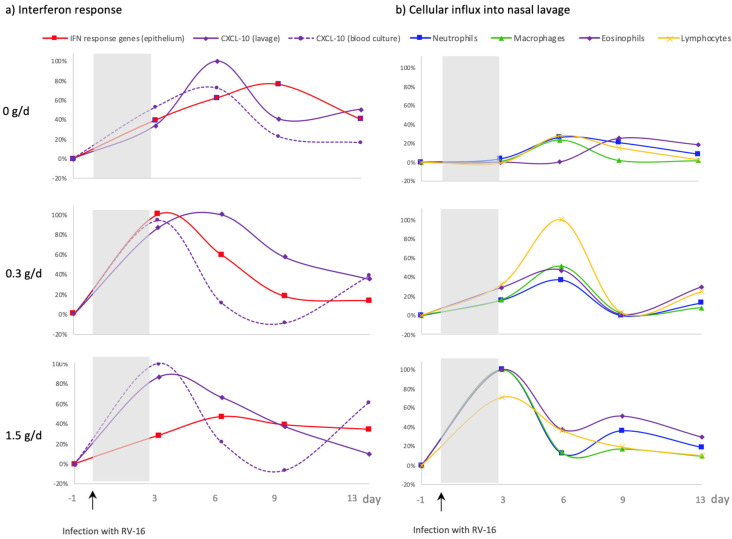
Kinetics of interferon responses and cellular influx after RV-16 infection (↑) expressed as change vs. d-1 calculated as percent of maximum effect across the three treatment groups. (**a**) Interferon responses in the local (nasal) compartment (CXCL-10 and IFN response genes), and in the systemic compartment (CXCL-10) after ex vivo stimulation. (**b**) Recruitment of immune cells into the local (nasal) compartment. Shaded area indicates time between infection and first biological marker sampling point.

After RV16 infection, a cRG-I dose-dependent acceleration was observed in all the local and systemic biomarkers measured, with the exception of local IFN response gene expression. Transcriptome analysis of nasal epithelial cells showed a faster expression of critical interferon response genes in the cRG-I groups than in the placebo and this accelerated expression showed a parabolic dose–response relationship [13]. To better understand this dose–response relationship, the kinetics of local and systemic anti-viral IFN responses were further explored (Figure 7a) as well as the kinetics of immune cell recruitment into the nasal compartment (Figure 7b). To this end, values were normalized to the maximum response over the three groups for each parameter (maximum response set at 100%). IFN response gene expression in the epithelium of the nasal compartment, represented by z-scores, were fastest in the 0.3 g/d cRG-I group and peaked on d3. This response peaked on d6 for the 1.5 g/d cRG-I group and on d9 for the 0 g/d group [13]. IFN-induced CXCL-10 increases in the nasal compartment (nasal lavage) were also faster in the cRG-I supplemented groups than the placebo group. Noticeably, levels of local CXCL-10 were similar for both cRG-I doses on d3. While levels continued to increase in the 0.3 g/d group, they were already decreasing in the 1.5 g/d group by d6. The responsiveness of PBMC, determined by the release of CXCL-10, measured in whole blood following ex vivo stimulation with poly-IC, was also increased after the viral challenge in the groups supplemented with cRG-I. In the nasal lavage neutrophils were the predominant cell type for all groups in line with the notion that neutrophils are rapidly recruited in response to infections. Figure 7b shows a dose-dependent acceleration of the influx of neutrophils, macrophages, lymphocytes, and eosinophils in the cRG-I groups. There were also significantly more inflammatory cells in the NAL, during the first week, in the cRG-I groups, shown previously [13]. None of the local innate immune responses measured prior to RV16 infection (d-55 up until d-1) were affected by dietary supplementation with cRG-I (data not shown).

## 4. Discussion

We previously showed the protective prophylactic effect of dietary supplementation with cRG-I. Daily intake prior to and during an RV16 infection accelerates and augments early innate immune and anti-viral responses and significantly reduces the severity and duration of common cold symptoms in healthy adults [13]. In this paper we show that supplementation with cRG-I also enhanced ex vivo immune responses of NK cells in blood, but not phagocytosis. In cRG-I supplemented groups, ex vivo stimulation of blood cells with poly-IC (a dsRNA analog) resulted in slightly more CXCL-10 prior to infection (NS) and a significant increase in CXCL-10 after infection. Production of other cytokines was not affected by cRG-I. Daily consumption of cRG-I also reduced the negative effects of a common cold on individual symptom scores and on quality of life attributes. 

### 4.1. More Pronounced Reduction in Severity and Duration of Common Cold Symptoms Following Dietary Supplementation with 0.3 g/d cRG-I

Even though subjects were only exposed to RV16 if they were symptom-free based on the Jackson questionnaire and negative for a broad-spectrum respiratory virus PCR, some subjects in the initial ITT analysis did have WURSS-21 symptom scores higher than zero on the day before RV16 infection. This could be attributed partly to a positive score on the question “Feeling tired” which does not feature in the Jackson score. Interestingly, a post hoc analysis excluding data from the subjects with a WURSS symptom score higher than zero prior to RV16 infection demonstrated even more clearly the beneficial effect of dietary supplementation with 0.3 g/d cRG-I on symptoms with a 33% reduction in severity and a 43% reduction in duration of symptoms. The dose of rhinovirus used in this study was purposefully chosen to model common cold symptoms that are relevant but not too severe. In the current study, the infection generally resulted in very mild symptoms, with total scores averaging 10 on a 70-point scale (range 0 to 54), but it is important to note that the infection did reflect a natural common cold infection.

### 4.2. Dietary Supplementation with cRG-I Dose Dependently Accelerates Anti-Viral Responses

It is striking that all local biological responses to RV16 were dose dependently affected by cRG-I supplementation, with the exception of the local IFN response. The acceleration of IFN gene expression was fastest in the 0.3 g/d group, and this bell-shaped dose–response was also reflected in the beneficial effects of cRG-I on symptoms. Notably, levels of CXCL-10 after RV16 infection were similar for both cRG-I groups on d3 while the influx of immune cells into the nasal compartment, in part a response to CXCL-10 release, was faster and higher in the 1.5 g/d group. Considering that CXCL-10 is likely consumed during the recruitment of immune cells, the IFN response and CXCL-10 release into NAL in the high dose group may have been earlier (d1 and/or d2) and possibly higher enabling a faster and stronger influx of immune cells in the 1.5 g/d group [41]. The rapid induction of ISGs is critical for the initiation of a protective response, just as a controlled down regulation of these responses is essential to maintain homeostasis and avoid excessive inflammation and collateral tissue damage. Rapid IFN responses to viral infections and subsequent expression of ISGs restricts viral replication and initiates innate immune defenses. It has been suggested that minimal expression levels of ISGs in the early stage of an infection protect the host against viral infection while higher levels are needed at later time points, in addition to other pathways, to resolve inflammation and initiate tissue repair [42]. Additionally, the proteins encoded by many ISGs act as pro-inflammatory mediators, but also control anti-viral and inflammatory responses [30,41]. Since the perception of symptom severity and duration is not determined by viral load, but rather by the magnitude of the hosts immune response to an infection and the speed of induction and resolution of that response [33] this may explain why we observed an optimal beneficial effect on perceived symptoms at 0.3 g/d. In any case, it is reassuring that cRG-I at 1.5 g/d did not lead to overstimulation of the immune system because biological feedback mechanisms maintained responsiveness within the normal range [18]. This resilience of the immune system, or maintenance of homeostasis, reflects immune fitness and a healthy state of wellbeing. Future intervention studies with cRG-I should also include earlier sampling points, for example on d1 and d2 for better temporal analysis of the interferon responses, as well as additional immune markers to better understand the effect of cRG-I on the resolution phase of the inflammatory response. 

### 4.3. Significant Benefit on Wellbeing during Common Cold Infection with cRG-I Dietary Supplementation

The host response to an RV infection is critical to curb the infection but also contributes to the development of clinical symptoms that can have a negative impact on the quality of life of the infected individual. The comprehensive set of questions in the validated WURSS-21 questionnaire was used to assess the impact of dietary supplementation with cRG-I on individual symptoms and daily activities following a mild RV16 infection. Supplementation with cRG-I showed a remarkably consistent and significant benefit on the subjective symptom scores, reducing severity and/or duration of most symptoms. The strongest effects were observed in the 0.3 g/d cRG-I group with subjects reporting more than 30% reduction in severity and duration of a runny nose and scratchy throat, and a 20–30% reduction in severity and duration of a sore throat and reduction in cough severity. Subjects in both cRG-I groups reported that their cold interfered more with their ability to breathe easily, and they reported more severe head and chest congestion symptoms. These effects were strongest in the 1.5 g/d group which is possibly related to a modulating effect of cRG-I on mucus production during the infection and might be explained by the faster infiltration of inflammatory cells (predominantly neutrophils) in the nasal cavity. Neutrophils play a role in mucus hypersecretion, which is also a normal response to RV infections and functions to clear the virus and damaged cells from the respiratory tract [43,44]. Additionally, temporary disruption of mucociliary clearance during the infection might contribute to this effect [45,46]. Interestingly, the beneficial effect of cRG-I on individual symptom scores was lower in the 1.5 g/d cRG-I group and might be attributed to the temporal down regulation of local IFN responses combined with the stronger influx of neutrophils [13].

**Figure 8:**
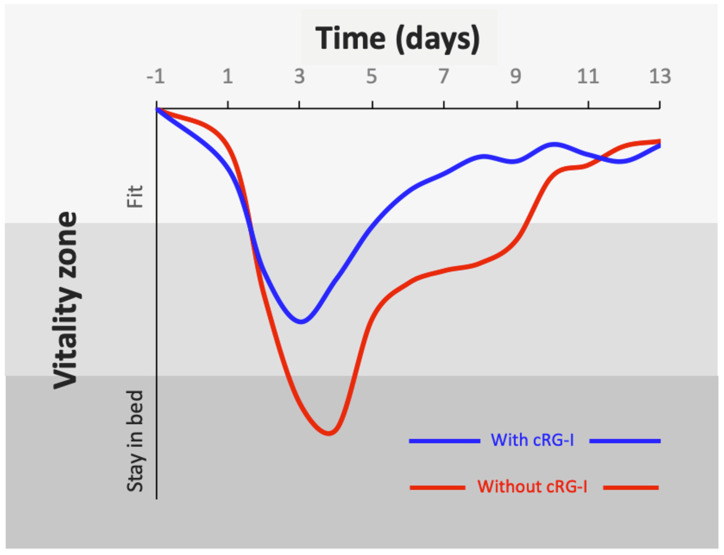
Exposure to an (experimental) rhinovirus infection initiates innate anti-viral and immune responses, which is accelerated by prior consumption of 0.3 g/d cRG-I. Inversion of the WURSS-21 score (score = zero on d-1), expressed conceptually as a “vitality zone”, demonstrates the mitigating effect of 0.3 g/d cRG-I on vitality in response to the impact of a common cold infection (infection on d0), thus visualizing the increased resilience to a respiratory infection.

Dietary supplementation with cRG-I also reduced the negative impact of a common cold on quality of life. A significant reduction was observed in the severity and duration of the negative impact of the RV16 infection on specific attributes such as walking, climbing stairs and exercise, accomplishing daily activities, and working inside and outside the home, as well as a significant reduction in the duration of the cold interfering with ability to think clearly and living your personal life. Clearly, RV infections have a negative impact on overall fitness and wellbeing, and dietary supplementation with 0.3 g/d cRG-I significantly ameliorated these negative effects. This can be conceptualized by inverting the overall WURSS-21 scores and plotting the data in terms of their impact on vitality (Figure 8). Minimizing the impact of common cold viruses on quality of life can potentially ameliorate the loss of productivity which accounts for millions of missed workdays and a significant burden on the economy, not to mention the impact of other acute respiratory infections. 

### 4.4. Dietary Intake of cRG-I Leads to Innate Immune Priming or Training

We have previously postulated that dietary supplementation with cRG-I exerts its beneficial effect on local anti-viral responses in the respiratory tract by directly modulating responsiveness of the innate immune system via cells (macrophages and DC) that sample the luminal content of the gut, and indirectly via modulating gut microbiota composition and production of metabolites including SCFA [7,13]. Studies using in vitro cultures of human blood and isolated PBMC have shown that cRG-I has the potency to modulate various aspects of immune function, including stimulation of NK cell and phagocyte activity, and modulation of cytokine production. The current data demonstrate that dietary supplementation with cRG-I augmented NK cell responsiveness prior to RV16 infection. Expression of CD107a following ex vivo incubation with K562 target cells was enhanced after 8 weeks supplementation in both cRG-I groups, with a more pronounced effect in the 0.3 g/d group. Activation of NK cells is strongly dependent on the presence and levels of cytokines such as IL-2 and type I IFNs [47]. Regulation of ISGs, mentioned above, may thus also contribute to the fact that the 0.3 g/d dose was the most potent modulator of NK activity. Ex vivo production of CXCL-10 in whole blood cultures stimulated with a canonical viral antigen poly-IC was somewhat increased on d-1 (prior to RV16 infection) compared to d-55 (NS) and was significantly higher on the first days after infection in both cRG-I groups. The ex vivo release of other cytokines was not significantly affected by cRG-I supplementation. These results indicate that dietary supplementation with cRG-I does not lead to broad, non-specific activation of the innate immune system, but rather enhances its responsiveness during immune challenges such as a viral infection. These and earlier findings support the notion that dietary intake of 0.3 g/d cRG-I leads to some form of priming or training of the innate immune system, and this altered responsiveness to an immune challenge can be observed in ex vivo NK cell activity and CXCL-10 production in the blood compartment and locally in the respiratory tract [3,7,11]. A limitation of this study is that NK cell and phagocytic activity were not measured after the RV16 challenge.

### 4.5. Dietary cRG-I Has a Dual Mechanism of Action

Next to the above-described direct effects on the innate immune system, we have previously shown in vitro that cRG-I is fermented by human fecal microbiota, inducing a shift in relative microbial community composition, and enhancing the production of SCFA, thereby revealing prebiotic properties [7,48,49]. This represents a second mechanism of action for cRG-I as recent studies with a variety of common viruses have demonstrated relationships between the microbiota and virus infections, and even microbiota-mediated protection against *Influenza A*, revealing an interplay between commensal and anti-viral interferon signaling pathways [50]. We are currently analyzing fecal samples that were collected during this study to explore possible relationships between microbiota, immune responses, and common cold symptoms (manuscript in preparation).

### 4.6. Future Perspectives

Fast innate immune and anti-viral interferon responses are key for host defense against many pathogens, suggesting that the accelerated and augmented responses after dietary intake of cRG-I could also be relevant against other airway infections including corona viruses, such as SARS-CoV-2, which are positive-sense single-stranded RNA viruses (+ssRNA) similar to rhinovirus. SARS-CoV-2 and other respiratory viruses also activate the innate immune system through PRR signaling and activation of ISGs [51]. Moreover, SARS-CoV-2 has been reported to evade host defenses by muting local IFN responses, suggesting that prophylactic intake of cRG-I could be potentially beneficial through accelerated and augmented IFN responses as well as ‘trained immunity’ [52,53].

## 5. Conclusions

The prophylactic intake of 0.3 g/d cRG-I offers clinically relevant benefits when needed, i.e., during an immune challenge by an infection. The severity and duration of common cold symptoms due to a rhinovirus infection were attenuated in the group taking 0.3 g/day cRG-I while biological feedback mechanisms maintained responses within the physiological range in the group taking 1.5 g/d cRG-I. We now demonstrate that dietary supplementation with cRG-I also enhanced immune responsiveness of cells from the systemic compartment, measured ex vivo, and shown by increased NK cell function and increased responsiveness of whole blood to the TLR3 agonist poly-IC. In addition, daily intake of cRG-I ameliorated the negative effects of this common cold infection on wellbeing as assessed by individual symptom scores and quality of life attributes. Since early innate immune and anti-viral responses to respiratory infections depend on the initial local IFN responsiveness, dietary intake of cRG-I may also prove effective in limiting the negative impact of other respiratory viral infections. RG-I from carrot is a safe, sustainable, and economically viable solution that could easily be integrated into food products and dietary supplements that aim to support immune fitness and wellbeing. 

## Data Availability

The data presented in this study are available on any reasonable request from the corresponding author. The data may become publicly available at a later stage when technical quality of the data can be maintained and when time permits.

## Supplementary Materials

The following supporting information can be downloaded at: https://www.mdpi.com/article/10.3390/nu14204258/s1, Supplementary Methods, Figure S1: WURSS21 question “how sick do you feel today?”; Table S1: Phagocytic capacity.
