# Supplementary material for: Effects of Dietary Supplementation with Carrot-Derived Rhamnogalacturonan-I (cRG-I) on Accelerated Protective Immune Responses and Quality of Life in Healthy Volunteers Challenged with Rhinovirus in a Randomized Trial
Source: Nutrients. 2022 Oct 12;14(20):4258. doi: 10.3390/nu14204258 (PMC9607575; doi:10.3390/nu14204258)
Supplement: Supplementary file 1 [file nutrients-14-04258-s001.zip › nutrients-1931234- supplementary.pdf]

Supplementary Methods

Phagocytosis

Opsonize *Ecoli* with serum (for complement), or with heat inactivated serum (for IgG).

1. 500 μL FITC-labelled *Ecoli* (1 x $E10^9$/ml) + 100 μL serum
2. Incubate at 37C for 30 min
3. Centrifuge for 8 min at 2800 g
4. Remove supernatant and wash with PBS, then centrifuge for 8 min at 2800 g
5. Resuspend pellet in 0.5 mL PBS

Phagocytosis assay

1. 300 μL heparin blood (24 x $E10^5$ cells) + 24 x $E.10^5$ *Ecoli* (in 240 μL) and mix well
2. Incubate samples for 5, 15 and 30 min at 37C. Incubate negative controls at 4C (on ice).
3. Store samples on ice after incubation.
4. Add 100 μL cold trypan blue to each tube and mix
5. Wash cells twice with PBEA, centrifuge for 8 min at 2800 g
6. Remove supernatant, add 1ml 1 x ELB solution and incubate on ice for 30min, mix every 5 min
7. Centrifuge tubes for 8 min at 2800 g
8. Wash pellet with PBEA
9. Resuspend cells with 300 μL FACS flow buffer and measure with flow cytometer.

Supplementary data

Figure S1. WURSS21 question "how sick do you feel today?"

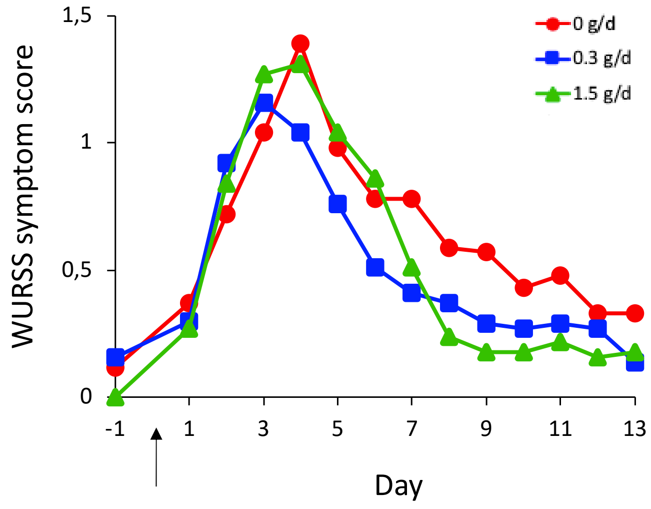

Figure S1. Time course of WURSS-21 question 1 "how sick do you feel today" (*p* < 0.0001).

Supplementary data

Table S1. Phagocytic capacity was evaluated in two distinct ways: 1) per interval of incubation (change between d-55 and d-1; incubation for 5, 15 and 30 minutes) and 2) per complete interval of incubation (change between d-55 and d-1; incubation 5 to 30 minutes), and measured as % of cells phagocytosing fluorescently labeled *Ecoli* and number of labeled bacteria in phagocytes (mean fluorescence intensity, MFI), in three opsonization conditions (IgG, IgG + complement, none).

Granulocytes % phagocytosis

		**Non -opsonized**						**IgG-opsonized**						**IgG+ complement-opsonized**					
		**5 min**		**15 min**		**30 min**		**5 min**		**15 min**		**30 min**		**5 min**		**15 min**		**30 min**	
**Day**	**N**	**Mean**	***SEM***	**Mean**	***SEM***	**Mean**	***SEM***	**Mean**	***SEM***	**Mean**	***SEM***	**Mean**	***SEM***	**Mean**	***SEM***	**Mean**	***SEM***	**Mean**	***SEM***
**0 g/day**																			
-55	60	1,3%	*0,3%*	11,3%	*0,9%*	32,8%	*1,3%*	1,8%	*0,3%*	20,0%	*1,3%*	46,6%	*1,5%*	1,5%	*0,3%*	17,6%	*1,1%*	44,4%	*1,5%*
-1	60	1,9%	*0,3%*	12,1%	*0,8%*	34,3%	*1,5%*	2,4%	*0,3%*	20,5%	*0,9%*	47,3%	*1,4%*	2,4%	*0,3%*	18,5%	*1,0%*	44,0%	*1,5%*
**0.3 g/day**																			
-55	58	2,0%	*0,3%*	13,0%	*1,2%*	36,0%	*1,6%*	2,5%	*0,4%*	23,7%	*1,3%*	50,0%	*1,7%*	2,1%	*0,3%*	19,4%	*1,1%*	44,5%	*1,5%*
-1	58	2,1%	*0,3%*	13,1%	*0,9%*	34,6%	*1,5%*	2,3%	*0,3%*	20,6%	*1,0%*	47,1%	*1,3%*	2,1%	*0,3%*	18,4%	*0,9%*	43,0%	*1,6%*
**1.5 g/day**																			
-55	59	1,8%	*0,3%*	12,9%	*1,0%*	38,6%	*1,6%*	2,9%	*0,4%*	22,2%	*1,4%*	50,0%	*1,7%*	1,9%	*0,3%*	18,5%	*1,2%*	45,3%	*1,7%*
-1	59	1,3%	*0,2%*	11,8%	*0,9%*	35,8%	*1,6%*	2,0%	*0,3%*	20,6%	*1,0%*	49,5%	*1,5%*	1,6%	*0,3%*	18,0%	*1,0%*	44,7%	*1,7%*

Monocytes % phagocytosis

		Non -opsonized						IgG-opsonized						IgG+ complement-opsonized					
		5 min		15 min		30 min		5 min		15 min		30 min		5 min		15 min		30 min	
**Day**	**N**	**Mean**	***SEM***	**Mean**	***SEM***	**Mean**	***SEM***	**Mean**	***SEM***	**Mean**	***SEM***	**Mean**	***SEM***	**Mean**	***SEM***	**Mean**	***SEM***	**Mean**	***SEM***
**0 g/day**																			
-55	60	1,7%	*0,3%*	7,0%	*0,6%*	14,2%	*0,9%*	1,5%	*0,2%*	9,8%	*0,7%*	19,9%	*0,9%*	1,5%	*0,3%*	9,4%	*0,6%*	18,7%	*0,8%*
-1	60	2,0%	*0,3%*	7,5%	*0,6%*	15,0%	*0,8%*	1,7%	*0,3%*	8,7%	*0,7%*	18,8%	*1,1%*	2,1%	*0,3%*	9,0%	*0,6%*	19,0%	*0,9%*
**0.3 g/day**																			
-55	58	2,2%	*0,4%*	8,5%	*0,8%*	17,7%	*1,0%*	2,2%	*0,3%*	12,1%	*0,7%*	21,8%	*1,2%*	2,1%	*0,3%*	10,0%	*0,7%*	19,3%	*1,1%*
-1	58	3,0%	*0,4%*	8,5%	*0,7%*	16,7%	*0,9%*	2,2%	*0,3%*	10,6%	*0,6%*	21,1%	*0,9%*	2,8%	*0,5%*	10,4%	*0,8%*	20,7%	*1,1%*
**1.5 g/day**																			
-55	59	2,3%	*0,5%*	8,7%	*0,8%*	17,8%	*1,2%*	2,4%	*0,4%*	11,4%	*1,0%*	22,3%	*1,3%*	2,0%	*0,5%*	9,8%	*0,8%*	21,0%	*1,1%*
-1	59	2,0%	*0,3%*	7,1%	*0,6%*	15,5%	*0,9%*	1,4%	*0,2%*	9,2%	*0,5%*	22,2%	*1,0%*	1,7%	*0,3%*	8,4%	*0,6%*	19,6%	*1,0%*

Granulocytes MFI

		Non -opsonized						IgG-opsonized						IgG+ complement-opsonized					
		5 min		15 min		30 min		5 min		15 min		30 min		5 min		15 min		30 min	
**Day**	**N**	**Mean**	***SEM***	**Mean**	***SEM***	**Mean**	***SEM***	**Mean**	***SEM***	**Mean**	***SEM***	**Mean**	***SEM***	**Mean**	***SEM***	**Mean**	***SEM***	**Mean**	***SEM***
**0 g/day**																			
-55	60	34	*8*	403	*40*	1559	*105*	52	*10*	783	*73*	2341	*152*	43	*11*	689	*60*	2197	*143*
-1	60	38	*6*	411	*29*	1550	*82*	62	*8*	780	*48*	2271	*122*	56	*7*	662	*41*	2025	*106*
**0.3 g/day**																			
-55	58	51	*10*	479	*55*	1737	*142*	73	*13*	970	*82*	2628	*178*	59	*10*	756	*64*	2200	*148*
-1	58	45	*7*	428	*31*	1554	*82*	57	*8*	795	*60*	2227	*124*	52	*8*	671	*48*	1945	*109*
**1.5 g/day**																			
-55	59	36	*7*	445	*41*	1813	*116*	71	*11*	874	*72*	2533	*172*	50	*10*	696	*59*	2218	*145*
-1	59	29	*6*	384	*29*	1559	*89*	51	*9*	757	*49*	2294	*111*	43	*9*	660	*47*	2006	*118*

Monocytes MFI

		Non -opsonized						IgG-opsonized						IgG+ complement-opsonized					
		5 min		15 min		30 min		5 min		15 min		30 min		5 min		15 min		30 min	
Day	N	Mean	*SEM*	Mean	*SEM*	Mean	*SEM*	Mean	*SEM*	Mean	*SEM*	Mean	*SEM*	Mean	*SEM*	Mean	*SEM*	Mean	*SEM*
**0 g/day**																			
-55	60	44	*7*	193	*20*	494	*34*	45	*7*	277	*25*	696	*49*	42	*9*	264	*21*	494	*34*
-1	60	43	*6*	199	*18*	505	*28*	41	*6*	226	*18*	629	*43*	53	*7*	226	*19*	505	*28*
**0.3 g/day**																			
-55	58	52	*13*	239	*26*	663	*53*	59	*8*	359	*30*	838	*65*	64	*13*	275	*26*	663	*53*
-1	58	69	*10*	203	*18*	546	*35*	53	*8*	286	*20*	704	*42*	64	*11*	255	*24*	546	*35*
**1.5 g/day**																			
-55	59	60	*16*	267	*32*	681	*70*	75	*15*	323	*33*	841	*77*	67	*18*	285	*27*	681	*70*
-1	59	47	*7*	186	*14*	475	*24*	37	*6*	228	*15*	719	*45*	39	*6*	230	*17*	475	*24*

The outcome showed that the longer the interval of incubation the higher the percentage of cells (either granulocytes or monocytes) having phagocytized *Ecoli* and the higher the MFI ($p < 0.001$); the batch of *Ecoli* used in the analyses demonstrated significant impacts on the outcome in percentage of cells (only granulocytes ($p < 0.03$) but not monocytes), but especially on MFI (both granulocytes and monocytes) ($p < 0.001$); the percentage of monocytes having phagocytized *Ecoli* was higher on d-55 than d-1 in the cRG-I groups ($p < 0.04$); the MFI was higher on d-55 than d-1, for both granulocytes and especially monocytes in the cRG-I groups ($p < 0.002$). Overall, cRG-I slightly but significantly (due to high power in the data) reduced the phagocytic capacity of granulocytes and monocytes.
